# Chemical Compositions and Enantiomeric Distributions of Foliar Essential Oils of *Chamaecyparis lawsoniana* (A. Murray bis) Parl, *Thuja plicata* Donn ex D. Don, and *Tsuga heterophylla* Sarg.

**DOI:** 10.3390/plants13101325

**Published:** 2024-05-11

**Authors:** Elizabeth Ankney, Kathy Swor, Ambika Poudel, Prabodh Satyal, Joyce Kelly R. da Silva, William N. Setzer

**Affiliations:** 1 Independent Researcher, 141 W. 17th St., Lafayette, OR 97127, USA; 2 Independent Researcher, 1432 W. Heartland Dr., Kuna, ID 83634, USA; 3 Aromatic Plant Research Center, 230 N 1200 E, Suite 100, Lehi, UT 84043, USApsatyal@aromaticplant.org (P.S.); 4 Laboratório de Biotecnologia de Enzimas e Biotransformações, Universidade Federal do Pará, Belém 66075-110, Brazil; joycekellys@ufpa.br; 5 Department of Chemistry, University of Alabama in Huntsville, Huntsville, AL 35899, USA

**Keywords:** Port Orford cedar, western red cedar, Cupressaceae, western hemlock, Pinaceae, gas chromatography, chiral

## Abstract

As part of our continuing interest in the essential oil compositions of gymnosperms, particularly the distribution of chiral terpenoids, we have obtained the foliar essential oils of *Chamaecyparis lawsoniana* (two samples), *Thuja plicata* (three samples), and *Tsuga heterophylla* (six samples) from locations in the state of Oregon, USA. The essential oils were obtained via hydrodistillation and analyzed by gas chromatographic techniques, including chiral gas chromatography—mass spectrometry. The major components in *C. lawsoniana* foliar essential oil were limonene (27.4% and 22.0%; >99% (+)-limonene), oplopanonyl acetate (13.8% and 11.3%), beyerene (14.3% and 9.0%), sabinene (7.0% and 6.5%; >99% (+)-sabinene), terpinen-4-ol (5.0% and 5.3%; predominantly (+)-terpinen-4-ol), and methyl myrtenate (2.0% and 5.4%). The major components in *T. plicata* essential oil were (−)-α-thujone (67.1–74.6%), (+)-β-thujone (7.8–9.3%), terpinen-4-ol (2.7–4.4%; predominantly (+)-terpinen-4-ol), and (+)-sabinene (1.1–3.5%). The major components in *T. heterophylla* essential oil were myrcene (7.0–27.6%), α-pinene (14.4–27.2%), β-phellandrene (6.6–19.3%), β-pinene (6.4–14.9%; >90% (−)-β-pinene), and (*Z*)-β-ocimene (0.7–11.3%). There are significant differences between the *C. lawsoniana* essential oils from wild trees in Oregon and those of trees cultivated in other geographical locations. The essential oil compositions of *T. plicata* are very similar, regardless of the collection site. There are no significant differences between *T. heterophylla* essential oils from the Oregon Coastal Range or those from the Oregon Cascade Range. Comparing essential oils of the Cupressaceae with the Pinaceae, there are some developing trends. The (+)-enantiomers seem to dominate for α-pinene, camphene, sabinene, β-pinene, limonene, terpinen-4-ol, and α-terpineol in the Cuppressaceae. On the other hand, the (−)-enantiomers seem to predominate for α-pinene, camphene, β-pinene, limonene, β-phellandrene, terpinen-4-ol, and α-terpineol in the Pinaceae.

## 1. Introduction

*Chamaecyparis* Spach is a genus in the Cupressaceae. The World Flora Online currently recognizes seven species of the genus [1], namely, *Chamaecyparis flifera* Veitch ex. Sénécl., *Chamaecyparis formosensis* Matsum. (Formosan cypress, endemic to Taiwan), *Chamaecyparis hodginsii* (Dunn) Rushforth (Po mu, found in eastern China and Vietnam), *Chamaecyparis lawsoniana* (A. Murray bis) Parl. (Port Orford cedar, found in western North America), *Chamaecyparis obtusa* (Siebold & Zucc.) Endl. (hinoki cypress, native to Japan), *Chamaecyparis pisifera* (Siebold & Zucc.) Endl. (Sawara cypress, native to Honshu and Kyushi, Japan), and *Chamaecyparis thyoides* (L.) Britton, Sterns & Poggenb. (Atlantic white cedar, found in the eastern United States) [2,3].

The genus *Thuja* L. (Cupressaceae) is represented by five taxa [4]: *Thuja koraiensis* Nakai (found in Jilin Province of China and in North and South Korea) [5], *Thuja occidentalis* L. (in eastern North America, the tree ranges from southeastern Canada, Minnesota, Michigan, and New England, south through the Appalachian Mountains) [6], *Thuja plicata* Donn ex. D. Don (two populations in western North America, a coastal population ranging from the Alaskan panhandle, coastal British Columbia south into coastal northern California, and an inland population found in the Rocky Mountains of British Columbia heading south to northern Idaho and western Montana) [7], *Thuja standishii* (Gordon) Carrière (native to Japan) [8], and *Thuja sutchuenensis* Franch. (native to Sichuan Province, China, but probably extinct in the wild due to deforestation) [9]. The genus has been important to the traditional healthcare systems in its natural ranges [10,11].

The genus *Tsuga* (Endl.) Carrière, in the family Pinaceae, is represented by five North American taxa, namely *Tsuga canadensis* (L.) Carrière (found in eastern North America), *Tsuga caroliniana* Engelm. (native to the Appalachian Mountains), *Tsuga heterophylla* (Raf.) Sarg. (found in western North America), *Tsuga mertensiana* (Bong.) Carrière (found in western North America), *Tsuga jeffreyi* (A. Henry) A. Henry (syn. *Tsuga mertensiana* subsp. *jeffreyi* (A. Henry) Silba) [12]; and seven East Asian species, *Tsuga chinensis* (Franch.) Pritz. (native to China, Taiwan, Tibet, and Vietnam), *Tsuga diversifolia* (Maxim.) Mast. (native to the Japanese islands of Honshū, Kyūshū, and Shikoku), *Tsuga dumosa* (D. Don) Eichler (native to the eastern Himalayas), *Tsuga forrestii* Downie (syn. *Tsuga chinensis* var. *forrestii* (Downie) Silba, found in the northeast Guizhou, southwest Sichuan, and northwest Yunnan provinces of China), *Tsuga sieboldii* (Siebold & Zucc.) Carrière (native to the Japanese islands of Honshū, Kyūshū, Shikoku, and Yakushima), *Tsuga thuja* A. Murray, and *Tsuga ulleungensis* G.P. Holman, Del Tredici, Havill, N.S. Lee & C.S. Campb. (endemic to Ulleungdo island, Korea) [13,14].

**Figure 1:**
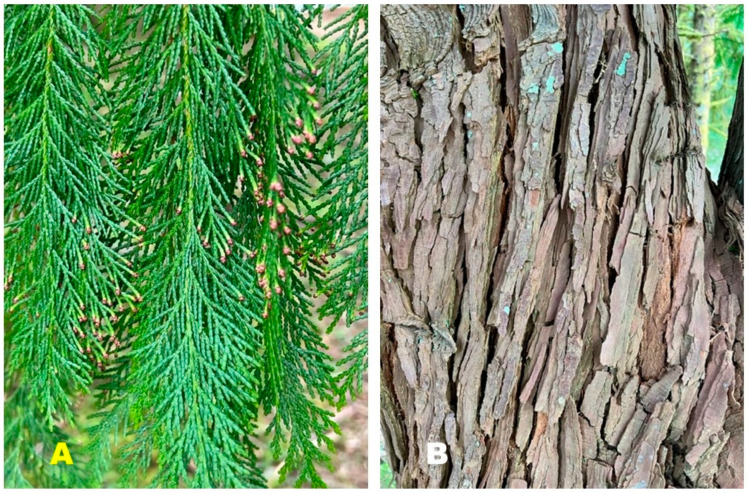
*Chamaecyparis lawsoniana* (A. Murray bis) Parl. (**A**): A photograph of its foliage, (**B**): A photograph of its bark (photographs taken by K. Swor at the time of sample collection).

**Figure 2:**
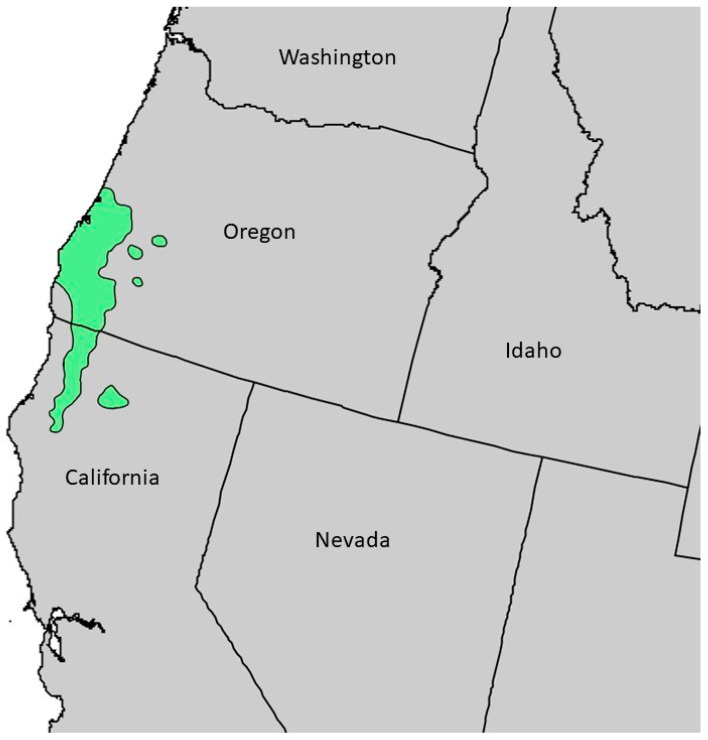
Natural range of *Chamaecyparis lawsoniana* [25]. This image is in the public domain in the United States because it only contains materials that originally came from the United States Geological Survey, an agency of the United States Department of the Interior.

*Chamaecyparis lawsoniana* (A. Murray bis) Parl., Cupressaceae (Port Orford cedar) is a large tree, around 50 m tall with a trunk up to 3 m in diameter [15]. The foliage has a lacy feathery appearance with leaves that are overlapping and scalelike, 2–3 mm long; the bark is thick, silvery-brown, and furrowed (Figure 1) [16]. The natural range of *C. lawsoniana* is limited to a small area of coastal Oregon into northern California (Figure 2) [17]. It has become an important ornamental outside of its natural range, particularly in Europe. Previous essential oil analyses have been carried out on *C. lawsoniana* cultivated in Japan [18], Belgium [19], Egypt [20,21], Iran [22], Spain [23], and Greece [24]. A purpose of the present study is to characterize the foliar essential oil of *C. lawsoniana* growing in its natural habitat in the Oregon Coastal Range.

**Figure 3:**
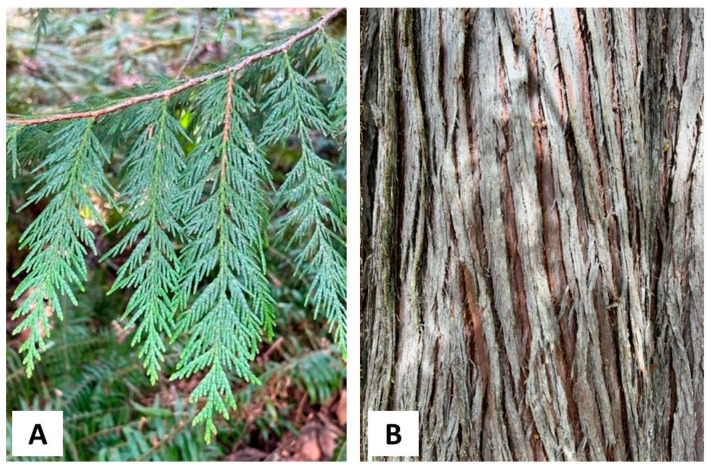
Photographs of *Thuja plicata* taken by K. Swor at the time of sample collection. (**A**): A photograph of its foliage. (**B**): A photograph of its bark.

**Figure 4:**
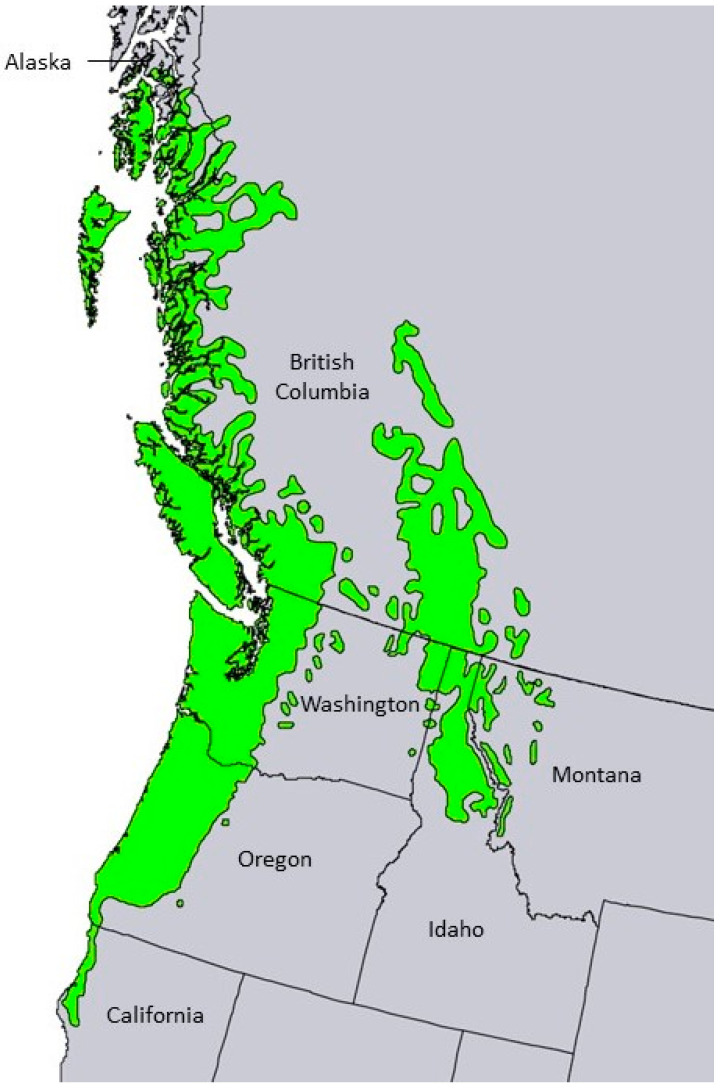
The native range of *Thuja plicata* [25]. This image is in the public domain in the United States because it only contains materials that originally came from the United States Geological Survey, an agency of the United States Department of the Interior.

*Thuja plicata* Donn ex D. Don, Cupressaceae (western red cedar) is a large tree, growing up to 75 m tall with a trunk up to 5 m in diameter; the thick, fibrous, fissured bark is reddish-brown or grayish-brown; the foliage is displayed as flat, pendant sprays with overlapping scale-like leaves (Figure 3) [26]. There are two separate ranges of *T. plicata*, a Coast–Cascade portion from southeastern Alaska (56°30′ N) to northwestern California (40°30′ N), and a Rocky Mountain section from British Columbia (54°30′ N) to Idaho and Montana (45°50′ N) (Figure 4) [26].

The heartwood of *T. plicata* has been shown to be a source of tropone monoterpenoids [27,28,29,30,31] and lignans [32,33,34,35,36,37,38,39,40], and the dilactone thujin [41], while the bark and aerial parts have yielded diterpenoid derivatives [42,43]. There have been several investigations on the foliar essential oil compositions of *T. plicata* growing wild in western North America [44,45], cultivated in Poland [46,47], cultivated in Serbia [48], and growing wild in Idaho, USA [49]. In addition, the volatiles from resin extracts of *T. plicata* cultivated in Czechia have been reported [50]. In this work, we had the opportunity to collect *T. plicata* samples from the Cascade Range of Oregon, so an additional purpose of this study is to test the hypothesis that the *T. plicata* from Oregon, a separate population from those from Idaho, presents differences in essential oil composition.

**Figure 5:**
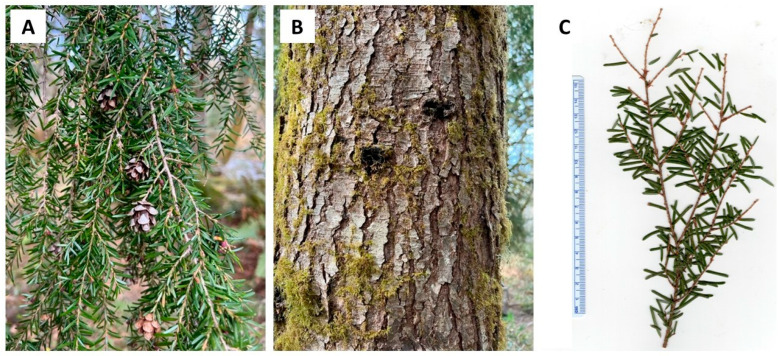
*Tsuga heterophylla* Sarg., Pinaceae (western hemlock). (**A**): Photograph of its foliage and cones. (**B**): Photograph of its bark. (**C**): Scan of the pressed foliage.

**Figure 6:**
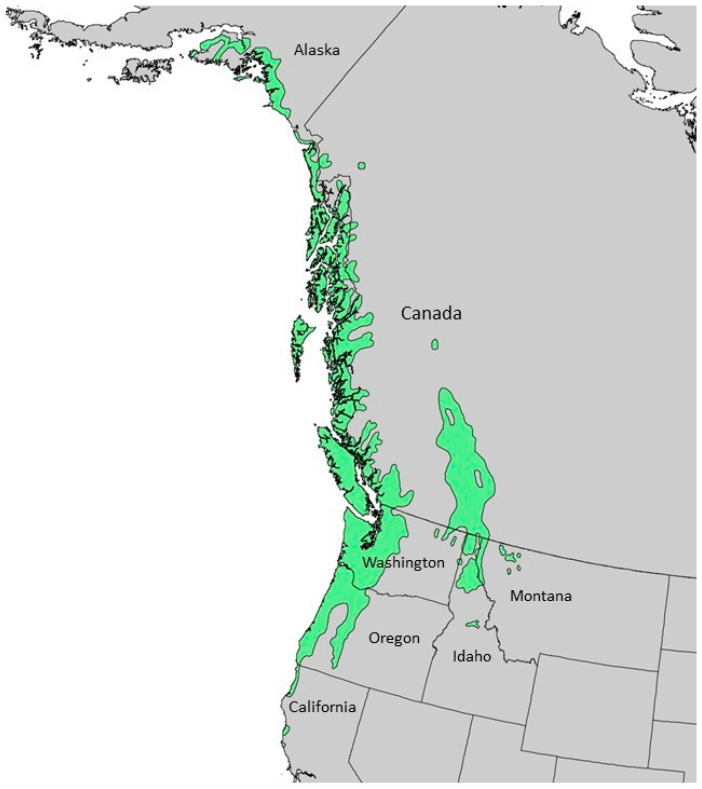
The native range of *Tsuga heterophylla* [25]. This image is in the public domain in the United States because it only contains materials that originally came from the United States Geological Survey, an agency of the United States Department of the Interior.

*Tsuga heterophylla* Sarg. (western hemlock) is a tree that grows up to 50 m tall with a trunk diameter up to 2 m; its leaves are needles, 5–20 mm long and 1.5–2 mm wide; its cones are small, 15–25 mm long and 10–25 mm wide; its bark is grey-brown, scaly, and moderately fissured (Figure 5) [51]. The native range of *T. heterophylla* is from the coast of southern Alaska, south through coastal British Columbia, Washington, Oregon, and into coastal northern California (Figure 6) [52]. The coastal range of *T. heterophylla* divides into an Oregon Coastal Range and a Cascade Range in Oregon. There is also a Rocky Mountain population that ranges from British Columbia south to northern Idaho and northwestern Montana (Figure 6).

Extracts of the wood of *T. heterophylla* have yielded lignans, including matairesinol [53], 8-hydroxy-α-conidendrin, 8-hydroxy-α-conidendric acid methyl ester [54], and 8-hydroxyoxomatairesinol [55]. Foliar volatiles have also been examined [56,57,58]. The purpose of the current study is to obtain foliar essential oils of *T. heterophylla* from both the Oregon Coastal Range and the Oregon Cascades to compare essential oil compositions from the two separated populations as well as to compare with compositions previously reported from British Columbia, Canada.

## 2. Results and Discussion

### 2.1. Essential Oil Compositions

#### 2.1.1. *Chamaecyparis lawsoniana*

The two *C. lawsoniana* pale yellow essential oils were obtained in 1.90% and 2.33% yields. Gas chromatographic analysis led to the identification of 136 components, which accounted for 97.3% and 96.8% of the total essential oil compositions (Table 1). The major components in the foliar essential oils were limonene (27.4% and 22.0%), oplopanonyl acetate (13.8% and 11.3%), beyerene (14.3% and 9.0%), sabinene (7.0% and 6.5%), terpinen-4-ol (5.0% and 5.3%), and methyl myrtenate (2.0% and 5.4%). There have been several reports on the essential oil compositions of *C. lawsoniana* cultivated outside the natural range of the tree, namely those cultivated in Japan [18], Belgium [19], Iran [22], Spain [23], Greece [24], and Egypt [20,21]. Not surprisingly, the essential oil compositions show wide variation, which can be attributed to the different geographical locations of these cultivated individuals.

**Figure 7:**
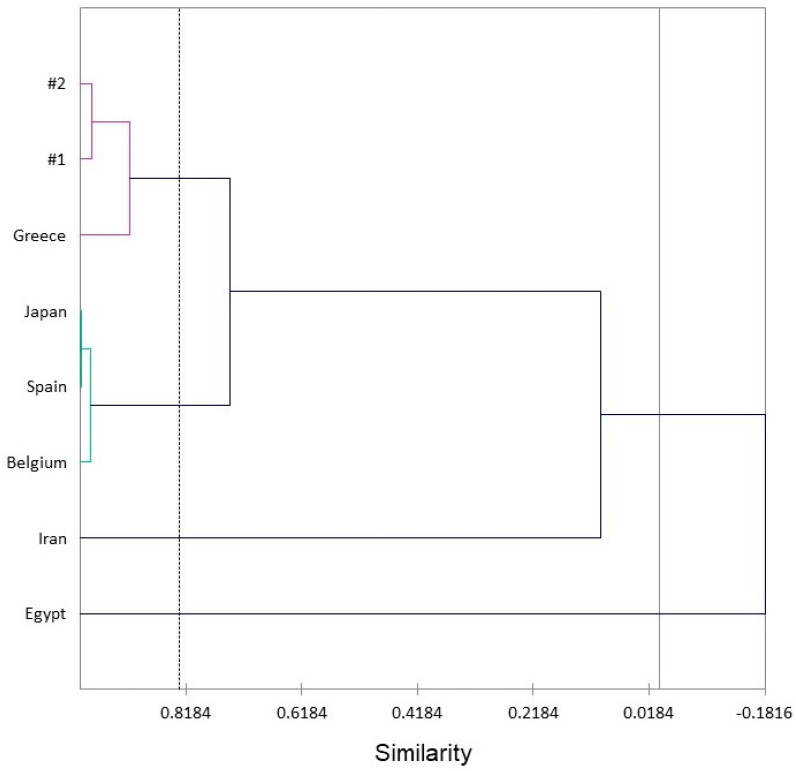
A dendrogram obtained via the hierarchical cluster analysis of *Chamaecyparis lawsoniana* essential oil compositions from different geographical locations: #1 and #2 (Oregon, this work), Greece [24], Japan [18], Spain [23], Belgium [19], Iran [22], and Egypt [21].

In order to visualize the differences in composition, a hierarchical cluster analysis (HCA) was carried out based on the percentages of the 30 most abundant components (Figure 7), and four clusters were identified. Cluster 1 grouped the cultivated sample, the sample from Greece, with the most remarkable similarity (91.6%) to the wild individuals due to the moderate amounts of limonene (18.5–27.4%), followed by oplopanonyl acetate (11.3–15.9%), and beyerene (9.0–17.1%). In addition, Cluster 1 showed a similarity of 74.3% with the samples from Spain, Belgium, and Japan (Cluster 2), which displayed high amounts of limonene as a major compound (57.6–77.7%). The individual cultivated in Iran (Cluster 3) was rich in *cis*-abienol (23.5%), *trans*-ferruginol (14.3%), α-cadinol (8.8%) and *cis*-muurola-4(14),5-diene (8.6%), while the sample from Egypt (Cluster 4) showed terpinen-4-ol (22.0%) and sabinene (21.0%) as significant compounds. For this reason, these samples displayed only slight similarity with the Oregon *C. lawsoniana* essential oils.

**Figure 8:**
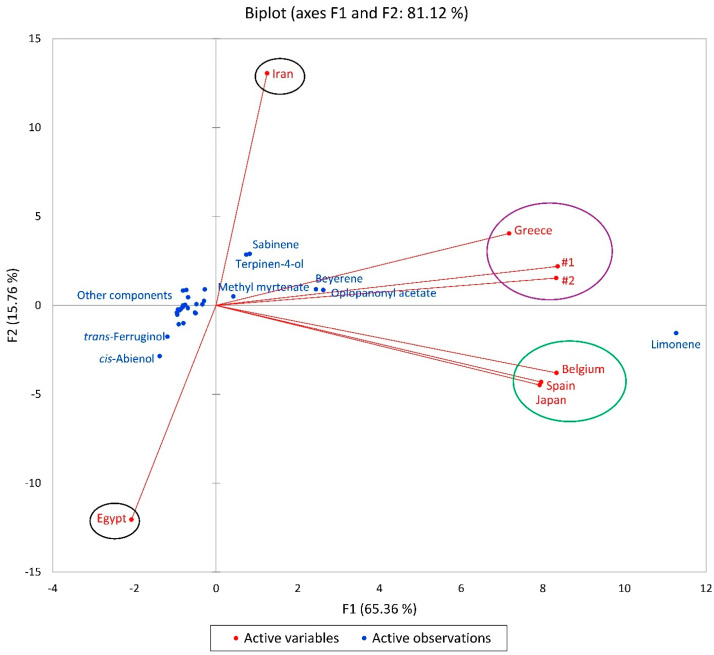
A biplot based on the principal component analysis (PCA) of *Chamaecyparis lawsoniana* essential oil compositions from different geographical locations: #1 and #2 (Oregon, this work), Greece [24], Japan [18], Spain [23], Belgium [19], Iran [22], and Egypt [21].

A principal component analysis (PCA) was applied to the constituents present to evaluate the chemical variety among the *C. lawsoniana* samples. The F1 and F2 of the constituents of oil samples explained 81.12% of the chemical variability, and the results corroborated the HCA analysis by grouping the samples into four main groups (Figure 8). Among the compounds with amounts above 5%, F1 showed positive correlations with limonene (11.267), oplopanonyl acetate (2.627), beyerene (2.448), sabinene (0.826), terpinen-4-ol (0.739), and methyl myrtenate (0.424), and negative correlations with *cis*-abienol (−1.380) and *trans*-ferruginol (−1.191). On the other hand, the F2 component explained 15.76% of the chemical variability, presenting positive correlations with sabinene (2.894), terpinen-4-ol (2.850), beyerene (0.909), γ-terpinene (0.898), oplopanonyl acetate (0.868) and camphor (0.830), and negative correlations with *cis*-abienol (−2.861), *trans*-ferruginol (−1.761), limonene (−1.562), α-cadinol (−1.064), and *cis*-muurola-4(14),5-diene (−1.006).

#### 2.1.2. *Thuja plicata*

The hydrodistillation of *T. plicata* foliage gave colorless-to-pale-yellow essential oils in yields of 0.76–1.03%. Gas chromatographic analysis led to the identification of 91 components, which accounted for 98.7% of the composition in each sample (Table 2). The major components in the essential oils were α-thujone (67.1–74.6%), β-thujone (7.8–9.3%), terpinen-4-ol (2.7–4.4%), and sabinene (1.1–3.5%).

**Figure 9:**
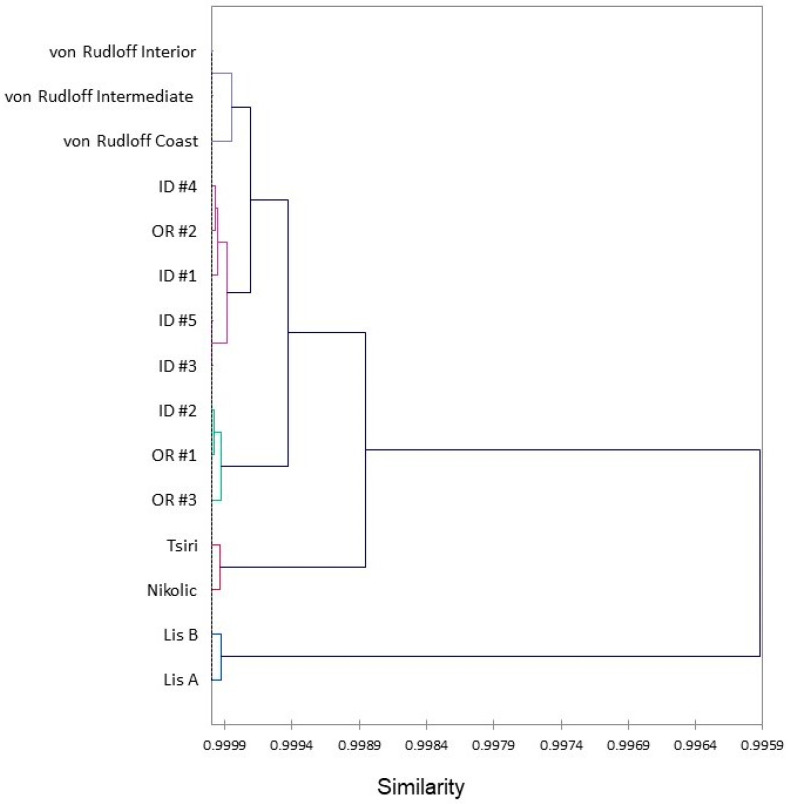
A dendrogram obtained via the hierarchical cluster analysis of *Thuja plicata* foliar essential oil compositions. von Rudloff [44], ID = samples from Idaho [49], OR = samples from Oregon (this work), Tsiri [46], Nikolic [48], Lis [47].

**Figure 10:**
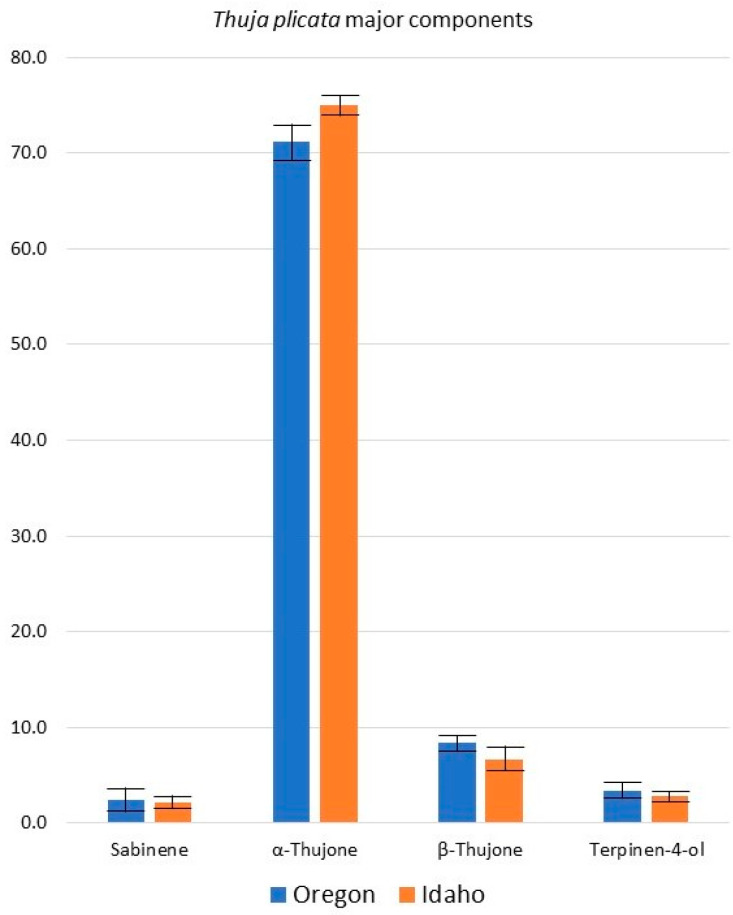
A comparison of the percentages of the major components of *Thuja plicata* foliar essential oils from Oregon and from Idaho. *t*-test *p*-values: Sabinene, *p* = 0.746; α-Thujone, *p* = 0.241; β-Thujone, *p* = 0.057; Terpinen-4-ol, *p* = 0.372.

The essential oil compositions of the Oregon *T. plicata* samples were very similar to those from our previous collection from northern Idaho [49] as well as those reported by von Rudloff and co-workers [44], Tsiri and co-workers [46], Lis and co-workers [47], and Nikolić and co-workers [48]. Indeed, a hierarchical cluster analysis (HCA) reveals very high similarity between the samples (Figure 9). The cluster analysis shows the greatest similarity, not surprisingly, between the samples from western North America (>99.94% similarity). Even the samples cultivated in Poland [47] showed >99.59% similarity to the North American samples. In comparing the Oregon samples from this work with those of our previous investigation of samples from Idaho, the concentrations of the major components are not statistically different (*t*-test, *p* > 0.05) (Figure 10). However, the β-thujone *t*-test showed a *p*-value of 0.057. In retrospect, the similarities in essential oil compositions are consistent with the genomic analysis of *T. plicata*; there is little genetic differentiation in this species [64,65,66].

**Figure 11:**
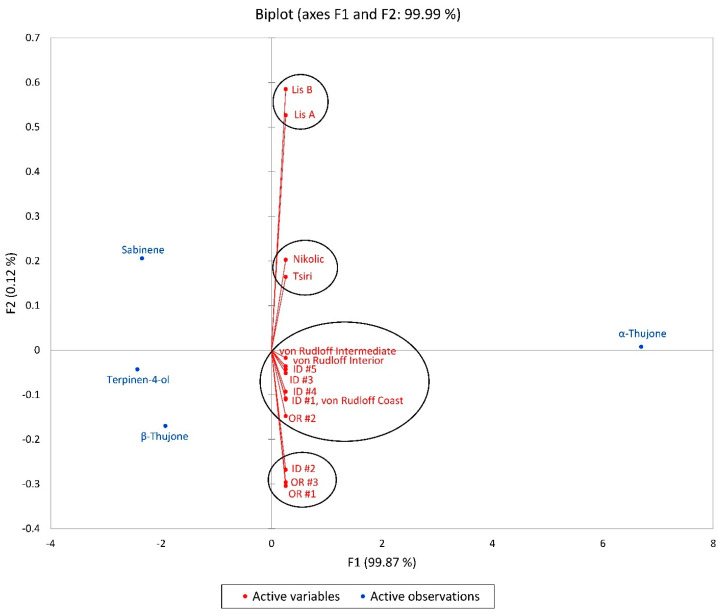
A biplot based on the principal component analysis (PCA) of *Thuja plicata* foliar essential oil compositions. von Rudloff [44], ID = samples from Idaho [49], OR = samples from Oregon (this work), Tsiri [46], Nikolic [48], Lis [47].

The *T. plicata* samples were subjected to principal component analysis (PCA) to understand their chemical variability comprehensively. The results, which are highly precise, revealed that F1 and F2 accounted for a significant 99.99% of the entire chemical variability. This analysis effectively grouped the samples into four main categories, as illustrated in Figure 11. F1 demonstrated a positive correlation with α-thujone (6.695), while terpinen-4-ol (−2.430), sabinene (−2.344), and β-thujone (−1.921), showed negative correlations. On the other hand, F2 had a positive correlation with sabinene (0.206), but a negative correlation only with β-thujone (−0.170). Notably, the samples collected in Oregon and those from Idaho and von Rudloff were found to have similar chemical characteristics, with α-thujone concentrations close to 70% and sabinene amounts of less than 5.0%.

#### 2.1.3. *Tsuga heterophylla*

The hydrodistillation of the foliage from the six *T. heterophylla* trees yielded colorless essential oils with yields of 5.28% to 7.75%. Monoterpene hydrocarbons dominated the essential oils of the *T. heterophylla*, with myrcene (7.0–27.6%), α-pinene (14.4–27.2%), β-phellandrene (6.6–19.3%), β-pinene (6.4–14.9%), and (*Z*)-β-ocimene (0.7–11.3%) as major products (Table 3). The diterpene beyerene (0.2–9.1%) and benzoic acid (1.7–4.7%) were also relatively abundant. Cascade Range samples *T.h.* #1, *T.h.* #2, and *T.h.* #3, and Coastal Range sample *T.h.* #5 were similar in composition to the samples from Maple Ridge, British Columbia, Canada, previously reported by von Rudloff [56].

**Figure 12:**
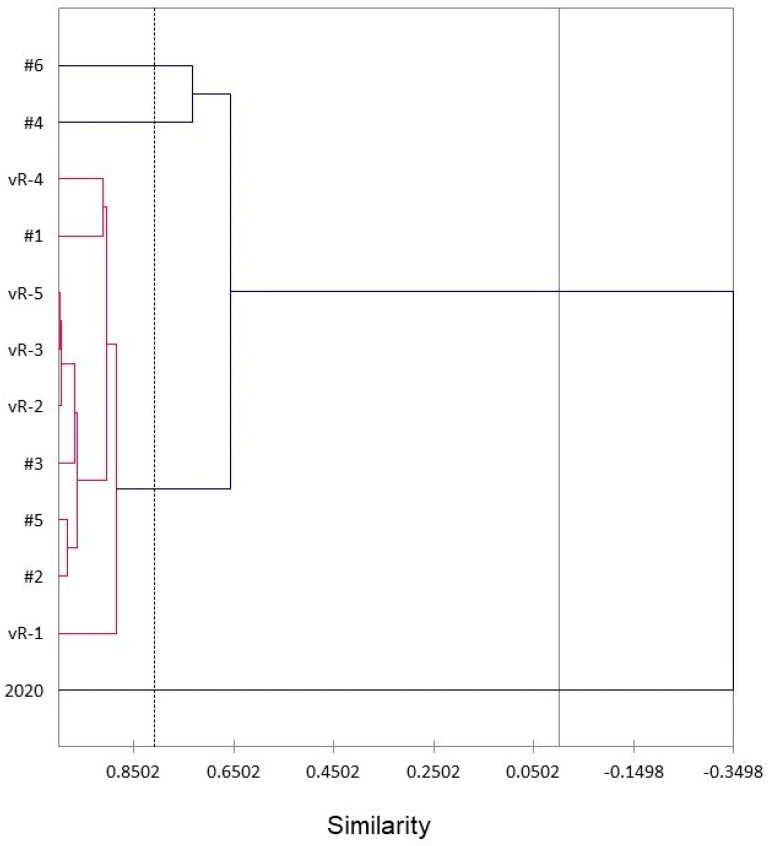
A dendrogram obtained via the hierarchical cluster analysis (HCA) of the major foliar essential oil components of *Tsuga heterophylla*. #1–#6, samples from Oregon (this work); vR-1–vR-5, von Rudloff, 1975 [56]; 2020, sample from Oregon collected in 2020, Ankney et al., 2021 [58].

A hierarchical cluster analysis (HCA) was carried out to visualize the similarities between the *T. heterophylla* essential oil compositions (Figure 12). The HCA shows that the British Columbia samples and the Oregon samples #1–#3 and #5 form a relatively large cluster with >88% similarity. Oregon Coastal Range samples #4 and #6 are qualitatively similar to the large cluster, but different (with 66% similarity) in that sample #4 showed a lower myrcene concentration (only 7.0%), while sample #6 showed a relatively low β-pinene concentration (6.4%); both samples were also low in β-phellandrene (6.6% and 8.4%, respectively). Curiously, a sample collected in 2020 from a single tree growing in the Hoyt Arboretum near Portland, Oregon, was very different in composition with only 5.7% monoterpene hydrocarbons, including no observed α-pinene [58]. The concentration of α-terpineol (10.3%) was relatively high in the Hoyt Arboretum sample. It is not clear what factors may account for the dissimilarity between the Hoyt Arboretum sample and the other *T. heterophylla* samples. The Hoyt Arboretum sample was collected in September 2020, while the samples in this present study were collected in April 2023. However, von Rudloff sampled trees from Vancouver, British Columbia, in both March 1974, and October 1974, which showed no significant difference in the α-pinene concentrations (both 15.3%) or the α-terpineol concentrations (0.8% and 0.5%, respectively) [56].

**Figure 13:**
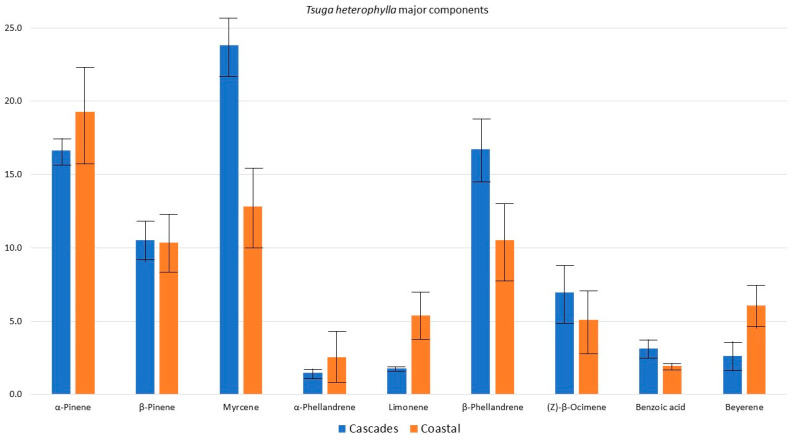
A comparison of the mean percentages of the major components of the *Tsuga heterophylla* foliar essential oils from the Oregon Cascade Range and the Oregon Coastal Range. *t*-test *p*-values: α-Pinene, *p* = 0.590; β-Pinene, *p* = 0.956; Myrcene, *p* = 0.068; α-Phellandrene, *p* = 0.616; Limonene, *p* = 0.175; β-Phellandrene, *p* = 0.219; (*Z*)-β-Ocimene, *p* = 0.594; Benzoic acid, *p* = 0.295; Beyerene, *p* = 0.173.

In comparing the compositions of the samples from the Oregon Cascade and Coastal ranges, there are no significant differences between their major components (α-pinene, β-pinene, myrcene, α-phellandrene, limonene, β-phellandrene, (*Z*)-β-ocimene, benzoic acid, and beyerene) (Figure 13). This result is consistent with the previous study by von Rudloff [56], who found no significant differences in the compositions of trees located in the British Columbia Coastal Range and those of trees from the Rocky Mountains. The low level of genetic diversity can be explained by past vegetation history. That is, genetic diversity in *T. heterophylla*, as well as *T. plicata*, is likely to be diminished due to a population bottleneck during the last glacial maximum [67].

**Figure 14:**
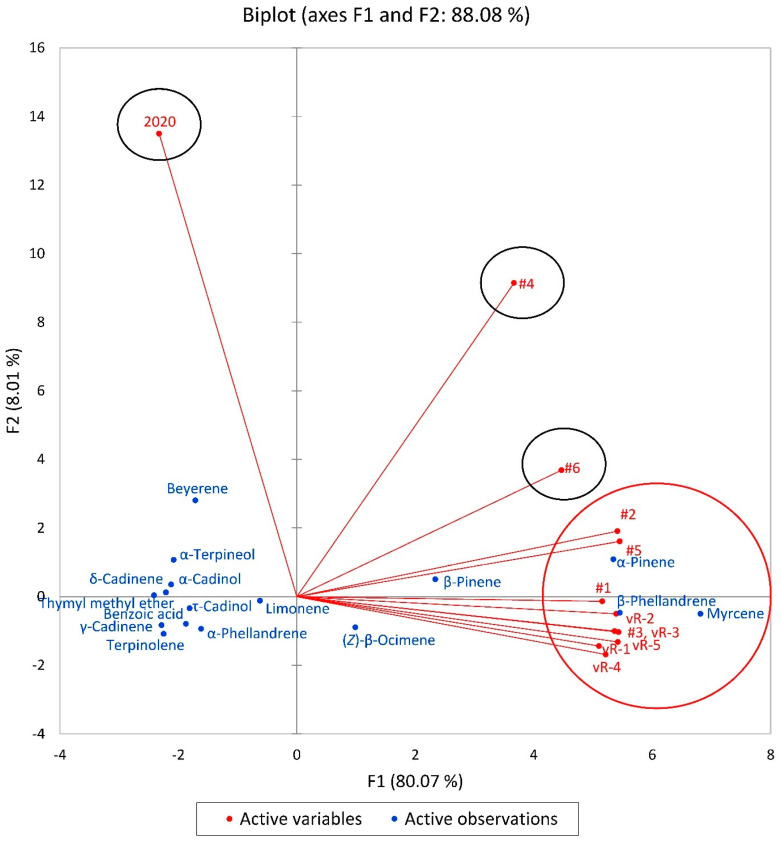
A biplot based on the principal component analysis (PCA) of the *Tsuga heterophylla* foliar essential oil compositions. #1–#6, samples from Oregon (this work); vR-1–vR-5, von Rudloff, 1975 [56]; 2020, sample from Oregon collected in 2020, Ankney et al., 2021 [58].

A PCA analysis of the *T. heterophylla* samples also was carried out and F1 and F2 explained a variability of 88.08%. The results observed corroborated the HCA analysis displaying a separation into four groups (Figure 14). The F1 samples showed positive correlations with samples rich in myrcene (6.818), β-phellandrene (5.459), α-pinene (5.347), β-pinene (2.342), and (*Z*)-β-ocimene (0.990), and negative correlations with thymyl methyl ether (−2.407), γ-cadinene (−2.280), terpinolene (−2.246), δ-cadinene (−2.207), α-cadinol (−2.120), α-terpineol (−2.078), benzoic acid (−1.868), τ-cadinol (−1.808), beyerene (−1.711), α-phellandrene (−1.612), and limonene (−0.620). On the other hand, F2 showed positive correlations with beyerene (2.804), α-pinene (1.087), and α-terpineol (1.072), but negative correlations with terpinolene (−1.086), α-phellandrene (−0.940), (Z)-β-ocimene (−0.895), γ-cadinene (−0.832), and benzoic acid (−0.796).

### 2.2. Enantiomeric Distribution

Enantioselective GC-MS analyses were carried out on the *C. lawsoniana*, *T. plicata*, and *T. heterophylla* foliar essential oils (Table 4, Table 5 and Table 6, respectively).

In *C. lawsoniana* (Table 4), (−)-α-thujene, (+)-*cis*-sabinene hydrate, (−)-bornyl acetate, and (+)-δ-cadinene were the only enantiomers detected. In addition, (+)-sabinene (enantiomeric excess, ee = 98.8 and 99.2%), (+)-limonene (ee = 99.6 and 99.8%), and (+)-*trans*-sabinene hydrate (ee = 89.9 and 92.6%) were the dominant enantiomers, while (+)-α-pinene (ee = 60.4 and 93.8%), (+)-terpinen-4-ol (ee = 45.0 and 34.4%), and (+)-α-terpineol (ee = 33.0 and 36.4%) were the major enantiomers in *C. lawsoniana* essential oil.

The exclusive enantiomers observed in *T. plicata* foliar essential oils (Table 5) include (−)-α-thujene, (+)-sabinene, (+)-β-pinene, (−)-α-thujone, and (+)-β-thujone. Although neither (−)-sabinene nor (−)-β-pinene were detected, a relatively large myrcene peak (RI = 1031) in the chiral GC-MS may have masked any (−)-sabinene or (−)-β-pinene, however. (+)-α-Pinene (95.5–99.0%), (+)-limonene (96.7–97.1%), (+)-*cis*-sabinene hydrate (96.8–97.6%), (+)-terpinen-4-ol (70.3–75.2%), and (+)-α-terpineol (60.6–64.5%) were also dominant enantiomers.

The enantioselective GC-MS of *T. heterophylla* essential oil (Table 6) showed α-pinene and linalool to be virtually racemic. The (+)-enantiomers were the predominant stereoisomers for sabinene, α-phellandrene, and (*E*)-nerolidol, while the (−)-enantiomers predominated for camphene, β-pinene, limonene, terpinen-4-ol, and α-terpineol; (−)-bornyl acetate, (−)-germacrene D, and (+)-δ-cadinene were the only enantiomers detected.

Based on this current work and previous studies of enantiomeric distributions of chiral monoterpenoids in conifer essential oils, there are some interesting trends (Table 7). (+)-α-Pinene is the dominant enantiomer in essential oils of the Cupressaceae, but, although it is not consistent, (−)-α-pinene generally predominates in the Pinaceae. Similar trends are seen for camphene, β-pinene, and limonene; the (−)-enantiomers are dominant in the Pinaceae while the (+)-enantiomers dominate the essential oils of the Cupressaceae. Although (+)-sabinene seems to be virtually exclusive in the Cupressaceae, the enantiomeric distribution is inconsistent in the Pinaceae. (−)-β-Phellandrene is clearly dominant in Pinaceae essential oils, but there are insufficient data to draw a conclusion regarding the Cupressaceae. (−)-Terpinen-4-ol and (−)-α-terpineol are slightly favored in the Pinaceae while the (+)-enantiomers are slightly favored in the Cupressaceae. There are not enough data regarding the enantiomeric distributions of linalool to draw a conclusion regarding the distribution trend.

## 3. Materials and Methods

### 3.1. Plant Material

The foliage of *C. lawsoniana* was collected from two separate trees (*C. lawsoniana* #1 and #2) on 15 April 2023, from the Van Duzer Forest, Oregon Coastal Range. The trees were identified in the field by W.N. Setzer using a field guide [16] and were verified through a comparison with samples from the New York Botanical Garden [74]. A voucher specimen (WNS-Cl-6886) has been deposited into the herbarium at the University of Alabama in Huntsville. The fresh foliage from each tree was frozen (−20 °C) and stored frozen until distillation. Foliage of *T. plicata* was collected from three different individual trees (*T. plicata* #1-#3) located near Mt. Hood Village, Oregon, on 14 April 2023 (Table 8). The trees were identified by W.N. Setzer [16,75] and a voucher specimen (WNS-Tp-6850) has been deposited into the herbarium at the University of Alabama in Huntsville. The fresh foliage was immediately frozen and stored frozen (−20 °C) until distillation. *Tsuga heterophylla* foliage from three individual trees (*T. heterophylla* #1-#3) was collected on 14 April 2023 near Mt. Hood Village, Oregon (Cascade Range) and from three individual trees (*T. heterophylla* #4-#6) on 16 April 2023 near Ross Lodge—Boger, Oregon (Coastal Range) (Table 8). The trees were identified in the field by W.N. Setzer using a field guide [16] and were verified through a comparison with botanical samples from the C. V. Starr Virtual Herbarium [76]. A voucher specimen, WNS-Th-6897, has been deposited into the herbarium at the University of Alabama in Huntsville. The foliage was frozen (−20 °C) and stored frozen until hydrodistillation.

### 3.2. Hydrodistillation

The fresh/frozen foliage of each sample was chopped and hydrodistilled for three hours using a Likens-Nickerson apparatus [77,78,79] with the continuous extraction of the distillate with dichloromethane (Table 8). Enough water to immerse the plant material was used for the hydrodistillation. The condenser was chilled (10–15 °C) using a refrigerated recirculating pump. Each plant sample was hydrodistilled once. The dichloromethane was evaporated using a stream of warm air.

### 3.3. Gas Chromatographic Analysis

The *C. lawsoniana*, *T. plicata*, and *T. heterophylla* foliar essential oils were analyzed via GC-MS, GC-FID, and chiral GC-MS as previously described [73]. The essential oil compositions were determined by comparing both MS fragmentation and RI values with those reported in the Adams [60], FFNSC3 [61], NIST20 [62], and Satyal [63] databases. The percent compositions were determined from raw peak areas (GC-FID) without standardization. Enantiomeric distributions were determined via the comparison of RI values with authentic samples (Sigma-Aldrich, Milwaukee, WI, USA), which were compiled in our in-house database.

### 3.4. Statistical Analyses

For the hierarchical cluster analysis (HCA) of *C. lawsoniana*, the eight essential oil compositions were treated as operational taxonomic units (OTUs), and the percentages of the 30 most abundant essential oil components (α-pinene, sabinene, myrcene, α-terpinene, limonene, γ-terpinene, camphor, terpinen-4-ol, citronellol, *p*-cymen-7-ol, methyl myrtenate, α-terpinyl acetate, 6-*epi*-β-cubebene, *cis*-cadina-1(6),4-diene, *cis*-muurola-4(14),5-diene, γ-amorphene, δ-cadinene, β-oplopenone, 1,10-di-*epi*-cubenol, τ-cadinol, α-cadinol, oplopanonyl acetate, beyerene, sandaracopimara-8(14),15-diene, manoyl oxide, abietatriene, *cis*-abienol, pimara-7,15-dien-3-one, *trans*-totarol, and *trans*-ferruginol) were used to describe the chemical associations between the *C. lawsoniana* essential oil samples. Pearson correlation was used to measure similarity, and the unweighted pair group method with arithmetic average (UPGMA) was used for cluster definition. The HCA analysis was carried out using XLSTAT v. 2018.1.1.62926 (Addinsoft, Paris, France). The HCA for *T. plicata* was carried out as described above using 15 essential oil compositions and the four most abundant essential oil components (sabinene, α-thujone, β-thujone, and terpinen-4-ol). The 16 major components of the *T. heterophylla* essential oils (α-pinene, β-pinene, myrcene, α-phellandrene, limonene, β-phellandrene, (*Z*)-β-ocimene, terpinolene, benzoic acid, α-terpineol, thymyl methyl ether, γ-cadinene, δ-cadinene, τ-cadinol, α-cadinol, and beyerene) were used to reveal the chemical associations between the 12 *T. heterophylla* essential oil samples as described above (Pearson correlation was used to measure similarity, and the UPGMA method was used for cluster definition).

Principal component analysis (PCA), type Pearson correlation, was carried out to verify the previous HCA analysis using the main essential oil components (as described above). The PCA analyses were carried out using XLSTAT v. 018.1.1.62926 (Addinsoft, Paris, France).

Student’s *t*-test [80,81] was used to evaluate the differences in the concentrations of sabinene, α-thujone, β-thujone, and terpinen-4-ol between the Oregon and the Idaho essential oil samples of *Thuja plicata*. Similarly, the *t*-test was used to compare the concentrations of α-pinene, β-pinene, myrcene, α-phellandrene, limonene, β-phellandrene, (*Z*)-β-ocimene, benzoic acid, and beyerene in the Coastal Range and Cascade Range essential oil samples of *Tsuga heterophylla*. Minitab^®^ v. 19.2020.1 (Minitab, LLC, State College, PA, USA) was used to carry out the *t*-tests.

## 4. Conclusions

The present work revealed that wild-growing native *Chamaecyparis lawsoniana* essential oils show significant differences compared to the essential oils from trees cultivated in other geographical locations. On the other hand, essential oils of *Thuja plicata* are very similar, regardless of the collection site. Likewise, there are no significant differences between the *Tsuga heterophylla* essential oils from the Oregon Coastal Range and those from the Oregon Cascade Range. Both *T. plicata* and *T. heterophylla* likely have diminished genetic diversity, likely due to population bottlenecks during the last ice age. An examination of the distribution of monoterpenoid enantiomers indicates that the (+)-enantiomers seem to dominate α-pinene, camphene, sabinene, β-pinene, limonene, terpinen-4-ol, and α-terpineol in the Cuppressaceae, while the (−)-enantiomers seem to predominate for α-pinene, camphene, β-pinene, limonene, β-phellandrene, terpinen-4-ol, and α-terpineol in the Pinaceae. It would be interesting to see if these trends in enantiomeric distributions continue with additional research on the essential oils of gymnosperms.

## Figures and Tables

**Table 1:** The foliar essential oil composition (%) of *Chamaecyparis lawsoniana* from the Oregon Coastal Range.

RI_calc_	RI_db_	Compounds	*C.l.* #1	*C.l.* #2
923	923	Tricyclene	tr	tr
925	925	α-Thujene	0.5	0.8
933	933	α-Pinene	0.4	2.3
947	948	α-Fenchene	tr	tr
949	950	Camphene	tr	tr
973	972	Sabinene	7.0	6.5
977	978	β-Pinene	tr	0.1
978	978	Oct-1-en-3-ol	0.1	0.2
989	989	Myrcene	1.7	1.6
1007	1006	α-Phellandrene	tr	tr
1017	1017	α-Terpinene	0.8	1.0
1025	1025	*p*-Cymene	0.2	0.6
1027	1026	2-Acetyl-3-methylfuran	-	tr
1030	1030	Limonene	27.4	22.0
1034	1031	β-Phellandrene	0.1	0.1
1034	1032	1,8-Cineole	tr	tr
1035	1034	(*Z*)-β-Ocimene	tr	tr
1045	1045	(*E*)-β-Ocimene	0.1	0.1
1058	1057	γ-Terpinene	2.2	2.7
1070	1069	*cis*-Sabinene hydrate	0.3	0.3
1085	1086	Terpinolene	0.7	0.7
1089	1090	Fenchone	tr	tr
1090	1091	*p*-Cymenene	tr	0.1
1101	1099	*trans*-Sabinene hydrate	0.2	0.2
1107	1105	α-Thujone	0.1	0.1
1113	1113	*p*-Mentha-1,3,8-triene	tr	tr
1116	1114	6-Camphenone	tr	tr
1118	1118	β-Thujone	tr	tr
1122	1120	*trans-p*-Mentha-2,8-dien-1-ol	0.1	0.1
1125	1124	*cis-p*-Menth-2-en-1-ol	0.3	0.3
1133	1134	*cis*-Limonene oxide	tr	-
1134	1135	2-Vinylanisole	tr	tr
1136	1137	*cis-p*-Mentha-2,8-dien-1-ol	0.1	0.1
1139	1139	Nopinone	tr	tr
1140	1136	*trans*-3-Caren-2-ol	tr	tr
1142	1142	*trans-p*-Menth-2-en-1-ol	0.2	0.2
1146	1145	*trans*-Verbenol	tr	tr
1147	1145	Camphor	0.1	0.1
1157	1157	Sabina ketone	-	tr
1169	1169	Umbellulone	tr	tr
1172	1170	Borneol	tr	tr
1176	1176	*cis*-Pinocamphone	tr	tr
1179	1179	2-Isopropenyl-5-methyl-4-hexenal	0.1	0.3
1181	1180	Terpinen-4-ol	5.0	5.3
1184	1188	Naphthalene	tr	-
1187	1186	*p*-Cymen-8-ol	0.1	0.2
1194	1196	(4*Z*)-Decenal	0.8	0.6
1195	1195	α-Terpineol	0.2	0.3
1196	1197	(4*E*)-Decenal	0.3	0.1
1197	1196	*cis*-Piperitol	0.1	0.1
1200	1201	*cis*-Piperitenol	tr	tr
1206	1206	Decanal	0.2	0.1
1207	1208	Verbenone	-	0.1
1209	1208	*trans*-Piperitol	0.1	0.1
1218	1218	*trans*-Piperitenol	tr	-
1219	1218	*trans*-Carveol	tr	-
1244	1246	Carvone	-	tr
1256	1258	(4*Z*)-Decen-1-ol	0.5	0.5
1259	1256	9-Decyn-1-ol	0.4	0.3
1272	1271	1-Decanol	tr	tr
1276	1276	Methyl nerate	0.2	0.1
1278	1277	Phellandral	tr	-
1284	1285	Bornyl acetate	0.7	0.9
1290	1289	Thymol	-	tr
1292	1293	Undec-2-one	tr	tr
1295	1294	Methyl myrtenate	2.0	5.4
1318	1318	(2*E*,4*E*)-Decadienal	0.1	0.2
1319	1319	Methyl geranate	tr	0.1
1324	1324	(2*E*,4*E*)-Decadien-1-ol	tr	0.1
1328	1327	*p*-Mentha-1,4-dien-7-ol	0.1	tr
1331	1332	*trans*-Carvyl acetate	tr	0.1
1346	1346	α-Terpinyl acetate	1.6	1.5
1357	1357	*cis*-Carvyl acetate	tr	tr
1374	1376	α-Ionol	tr	0.1
1377	1378	Geranyl acetate	tr	0.1
1393	1398	Cedr-8(15)-ene	0.1	0.1
1395	1393	Methyl perillate	0.1	0.1
1398	1401	Undec-10-enal	0.1	tr
1419	1417	(*E*)-β-Caryophyllene	0.1	-
1421	1421	(*E*)-α-Ionone	tr	tr
1423	1423	β-Cedrene	-	tr
1434	1433	*cis*-Thujopsene	0.3	1.0
1437	1437	*iso*-Bazzanene	tr	tr
1441	1440	Dihydro-β-ionol	0.1	0.1
1445	1446	*cis*-Muurola-3,5-diene	0.8	0.8
1455	1454	α-Humulene	0.1	tr
1459	1463	*cis*-Cadina-1(6),4-diene	0.2	0.2
1462	1463	*cis*-Muurola-4(14),5-diene	1.7	1.6
1464	1458	Sabinyl isovalerate	0.4	0.3
1474	1471	β-Acoradiene	0.1	-
1482	1480	Germacrene D	0.1	-
1492	1490	γ-Amorphene	0.2	0.2
1496	1496	*trans*-Muurola-4(14),5-diene	0.7	0.6
1497	1497	α-Muurolene	tr	tr
1503	1503	β-Himachalene	0.1	0.5
1505	1506	α-Chamigrene	tr	tr
1507	1505	Cuparene	tr	tr
1513	1512	γ-Cadinene	tr	tr
1515	1515	Cubebol	tr	tr
1519	1518	δ-Cadinene	1.5	1.8
1520	1519	*trans*-Calamenene	tr	tr
1531	1531	10-*epi*-Cubenol	0.9	0.7
1535	1535	γ-Cuprenene	0.1	0.2
1537	1538	α-Cadinene	tr	tr
1547	1548	*cis*-Muurola-5-en-4β-ol	0.4	0.2
1558	1558	*cis*-Muurola-5-en-4α-ol	0.4	0.3
1571	1571	(3*Z*)-Hexenyl benzoate	tr	0.1
1577	1576	Spathulenol	tr	0.1
1582	1587	Caryophyllene oxide	tr	tr
1604	1606	β-Oplopenone	0.8	1.2
1607	1599	Widdrol	0.1	0.1
1609	1606	Cedrol	0.4	1.4
1616	1614	1,10-di-*epi*-Cubenol	1.1	1.3
1642	1640	τ-Cadinol	0.3	0.4
1644	1644	τ-Muurolol	tr	0.1
1647	1643	Cubenol	0.1	0.2
1655	1655	α-Cadinol	0.5	0.6
1662	1664	*ar*-Turmerone	tr	0.1
1667	1668	β-Turmerone	0.1	0.1
1676	1673	Acorenone A	0.1	0.2
1684	1686	*epi*-α-Bisabolol	-	0.2
1706	1708	*cis*-Thujopsenol	0.1	0.1
1713	1715	*trans*-Thujopsenal	0.1	0.5
1733	1735	Oplopanone	0.1	0.1
1874	1875	Oplopanonyl acetate	13.8	11.3
1937	1933	Beyerene	14.3	9.0
1943	1941	Pimaradiene I	-	0.1
1963	1968	Sandaracopimara-8(14),15-diene	0.3	0.3
1991	1989	Manoyl oxide	0.2	0.5
1995	1997	Kaur-15-ene	0.2	0.1
2002	1998	Luxuriadiene	-	2.7
2037	---	Atis-16-ene	tr	tr
2043	2045	Kaur-16-ene	0.1	0.1
2050	2049	Abietatriene	0.1	0.1
2240	---	15-Beyeren-19-ol, methyl derivative	0.4	0.4
2299	2302	*trans*-Totarol	0.5	0.4
		Monoterpene hydrocarbons	41.2	38.5
		Oxygenated monoterpenoids	12.1	16.1
		Sesquiterpene hydrocarbons	6.0	6.9
		Oxygenated sesquiterpenoids	19.3	19.3
		Diterpenoids	16.1	13.6
		Benzenoid aromatics	tr	0.1
		Others	2.6	2.4
		Total identified	97.3	96.8

RI_calc_ = Retention index calculated with respect to a homologous series of *n*-alkanes on a ZB-5ms column [59]. RI_db_ = Reference retention index from the databases [60,61,62,63]. tr = trace (<0.05%).

**Table 2:** The foliar essential oil composition (percentages) of *Thuja plicata* from the Cascade Range, Oregon.

RI_calc_	RI_db_	Compounds	*T.p.* #1	*T.p.* #2	*T.p.* #3
842	842	Ethyl 2-methyl butyrate	0.1	0.1	0.1
924	925	α-Thujene	0.1	0.2	0.2
932	933	α-Pinene	0.4	0.6	0.7
946	948	α-Fenchene	tr	tr	tr
948	950	Camphene	tr	tr	tr
971	971	Sabinene	1.1	3.5	2.6
976	978	β-Pinene	tr	0.1	0.1
978	978	Oct-1-en-3-ol	tr	tr	tr
987	989	Myrcene	0.5	1.7	1.0
997	997	Ethyl hexanoate	tr	tr	tr
1016	1017	α-Terpinene	0.3	0.7	0.5
1020	1022	Ethyl 3-methylbut-3-enyl carbonate	tr	tr	tr
1024	1025	*p*-Cymene	0.6	0.3	0.5
1026	1026	2-Acetyl-3-methylfuran	tr	tr	tr
1028	1030	Limonene	0.5	0.9	0.6
1030	1031	β-Phellandrene	tr	tr	tr
1034	1037	5-Methyl-(5*E*)-octen-2-one	0.1	tr	0.1
1056	1057	γ-Terpinene	0.6	1.3	0.8
1070	1069	*cis*-Sabinene hydrate	0.3	0.4	0.2
1084	1086	Terpinolene	0.1	0.3	0.2
1090	1091	*p*-Cymenene	tr	tr	tr
1095	1093	Ethyl sorbate	tr	tr	tr
1098	1098	Perillene	tr	tr	0.1
1107	1105	α-Thujone	74.6	67.1	71.7
1120	1118	β-Thujone	8.0	7.8	9.3
1123	1122	*trans-p*-Mentha-2,8-dien-1-ol	tr	tr	tr
1125	1124	*cis-p*-Menth-2-en-1-ol	0.2	0.3	0.2
1127	1127	α-Campholenal	0.1	tr	tr
1139	1138	*trans*-Sabinol	0.1	tr	0.1
1141	1141	*trans*-Pinocarveol	tr	tr	tr
1142	1142	*trans-p*-Menth-2-en-1-ol	0.1	0.2	0.2
1145	1145	*trans*-Verbenol	0.1	0.1	0.1
1147	1145	Camphor	tr	tr	tr
1152	1153	*neo*-3-Thujanol	0.1	0.1	0.1
1157	1157	Sabina ketone	0.2	0.1	0.2
1169	1168	α-Phellandrene epoxide	tr	0.1	tr
1169	1169	Ethyl benzoate	tr	-	tr
1171	1171	*p*-Mentha-1,5-dien-8-ol	tr	tr	tr
1175	1176	*trans*-Isopulegone	0.1	0.1	0.1
1181	1180	Terpinen-4-ol	2.7	4.4	3.2
1187	1186	*p*-Cymen-8-ol	0.2	0.2	0.2
1192	1194	*p*-Mentha-1,5-dien-7-ol	0.1	tr	0.1
1195	1195	α-Terpineol	0.2	0.4	0.3
1198	1197	Methyl chavicol (=Estragole)	0.5	0.8	0.6
1207	1205	Verbenone	0.1	tr	0.1
1209	1208	*trans*-Piperitol	0.1	0.1	tr
1219	1218	*trans*-Carveol	0.1	tr	0.1
1238	1238	Carvacryl methyl ether	tr	tr	tr
1242	1242	Cuminal	tr	tr	0.1
1244	1242	Carvone	0.1	tr	tr
1246	1250	Ethyl (2*E*)-octenoate	tr	tr	tr
1249	1248	Carvotanacetone	0.1	-	0.1
1249	1249	Linalyl acetate	-	0.1	-
1260	1260	*trans*-Sabinene hydrate acetate	0.1	0.1	0.1
1268	1267	*neo*-3-Thujyl acetate	0.1	0.2	0.2
1282	1280	Phellandral	tr	-	tr
1283	1282	Bornyl acetate	0.1	0.1	0.1
1287	1286	*trans*-Sabinyl acetate	tr	0.1	0.1
1289	1293	3-Thujanyl acetate	0.1	0.1	0.1
1291	1290	Menthyl acetate	0.3	0.5	0.4
1292	1291	*p*-Cymen-7-ol	0.1	-	tr
1297	1300	Carvacrol	0.1	0.1	0.1
1315	1322	Myrtenyl acetate	0.1	0.1	0.1
1328	1327	*p*-Mentha-1,4-dien-7-ol	0.1	0.1	0.2
1335	1335	4-Terpinyl acetate	0.1	0.1	0.1
1345	1346	α-Terpinyl acetate	0.2	0.4	0.2
1348	1349	Citronellyl acetate	tr	tr	tr
1357	1361	Neryl acetate	-	tr	-
1380	1378	Geranyl acetate	0.3	0.4	0.4
1397	1395	Ethyl decanoate	tr	tr	tr
1402	1403	Methyl eugenol	tr	tr	tr
1424	1426	Cuminyl acetate	tr	tr	tr
1447	1448	(*E*)-Cinnamyl acetate	-	-	tr
1468	1467	β-Acoradiene	0.1	-	-
1497	1495	Tridecan-2-one	tr	tr	tr
1520	1520	δ-Cadinene	tr	0.1	tr
1581	1578	Furopelargone B	0.1	0.1	-
1604	1601	Longiborneol (=Juniperol)	-	-	0.1
1605	1607	β-Oplopenone	tr	0.1	tr
1657	1655	α-Cadinol	tr	0.2	0.1
1664	1664	*ar*-Turmerone	tr	tr	tr
1669	1668	α-Turmerone	0.1	tr	0.1
1734	1735	Oplopanone	0.1	0.1	0.1
1899	1896	Rimuene	1.0	0.8	0.3
1934	1931	Beyerene	0.9	0.6	0.4
1996	1997	Kaur-15-ene	tr	tr	tr
2038	---	Atis-16-ene	tr	tr	tr
2052	2049	Abietatriene	tr	tr	tr
2243	---	15-Beyeren-19-ol	0.5	0.5	0.5
2301	2315	*trans*-Totarol	0.2	0.2	0.1
2319	---	15-Beyeren-19-ol acetate	1.7	1.9	1.1
		Monoterpene hydrocarbons	4.2	9.6	7.1
		Oxygenated monoterpenoids	89.2	83.6	88.1
		Sesquiterpene hydrocarbons	0.1	0.1	tr
		Oxygenated sesquiterpenoids	0.3	0.4	0.3
		Diterpenoids	4.4	4.1	2.4
		Benzenoid aromatics	0.5	0.8	0.6
		Others	0.1	0.1	0.2
		Total identified	98.7	98.7	98.7

RI_calc_ = Retention index calculated with respect to a homologous series of *n*-alkanes on a ZB-5ms column [59]. RI_db_ = Reference retention index from the databases [60,61,62,63]. tr = trace (<0.05%).

**Table 3:** The foliar essential oil compositions (percentages) of the *Tsuga heterophylla* from the Oregon Cascade Range and the Oregon Coastal Range.

RI_calc_	RI_db_	Compounds	Cascade Range			Coastal Range		
			*T.h.* #1	*T.h.* #2	*T.h.* #3	*T.h.* #4	*T.h.* #5	*T.h.* #6
800	797	(3*Z*)-Hexenal	0.1	0.1	0.1	0.1	0.1	0.2
802	802	Hexanal	tr	0.1	tr	tr	0.1	0.1
849	849	(2*E*)-Hexenal	1.5	1.9	1.2	1.7	3.8	3.1
851	853	(3*Z*)-Hexenol	0.2	0.3	0.2	0.3	0.5	0.4
922	923	Tricyclene	0.1	0.1	0.1	0.1	0.1	0.1
925	925	α-Thujene	0.1	0.1	0.1	0.1	0.1	tr
933	932	α-Pinene	17.1	18.4	14.4	15.3	15.4	27.2
947	948	α-Fenchene	tr	tr	tr	tr	tr	tr
949	950	Camphene	0.3	0.3	0.2	0.3	0.2	0.3
972	972	Sabinene	0.1	0.1	0.2	0.3	0.4	0.2
977	978	β-Pinene	7.4	11.6	12.6	14.9	9.7	6.4
989	989	Myrcene	27.6	19.4	24.3	7.0	17.7	13.7
1005	1005	*p*-Mentha-1(7),8-diene	-	-	-	tr	-	-
1007	1007	α-Phellandrene	2.2	1.4	0.9	-	1.6	6.0
1009	1008	δ-3-Carene	0.1	0.1	tr	tr	tr	0.1
1017	1017	α-Terpinene	0.1	0.1	tr	-	0.1	0.1
1024	1025	*p*-Cymene	1.2	0.7	1.3	1.5	1.1	1.8
1029	1030	Limonene	1.7	1.8	1.9	7.2	1.9	7.0
1031	1031	β-Phellandrene	11.6	19.3	19.2	6.6	16.6	8.4
1033	1032	1,8-Cineole	-	-	-	tr	-	-
1035	1034	(*Z*)-β-Ocimene	11.3	4.3	5.2	0.7	8.0	6.6
1045	1045	(*E*)-β-Ocimene	0.4	0.2	0.1	tr	0.2	0.2
1050	1051	2,3,6-Trimethylhepta-1,5-diene	-	-	-	0.2	-	-
1057	1057	γ-Terpinene	0.1	0.3	0.1	-	0.2	0.1
1065	1068	Acetophenone	-	-	-	tr	-	-
1070	1069	*cis*-Sabinene hydrate	-	-	-	tr	-	-
1085	1086	Terpinolene	0.4	0.6	0.4	-	0.6	0.6
1090	1091	*p*-Cymenene	-	-	-	-	0.1	-
1090	1090	6,7-Epoxymyrcene	0.1	0.1	0.1	0.3	-	tr
1091	1091	Rosefuran	0.1	tr	0.1	0.1	tr	tr
1098	1098	Perillene	tr	tr	0.1	0.2	-	tr
1099	1101	Linalool	0.2	0.1	0.1	tr	0.1	0.1
1099	1101	α-Pinene oxide	-	-	-	0.6	-	-
1107	1105	α-Thujone	-	tr	-	-	-	-
1119	1119	*endo*-Fenchol	0.1	tr	0.1	0.1	0.1	tr
1124	1124	*cis-p*-Menth-2-en-1-ol	0.2	0.3	0.3	0.4	0.3	0.2
1126	1125	α-Campholenal	tr	tr	0.1	0.1	0.1	tr
1127	1127	(4*E*,6*Z*)-*allo*-Ocimene	0.4	0.1	0.2	-	0.3	0.2
1129	1129	(*Z*)-Myroxide	-	-	-	0.1	-	-
1132	1132	*cis*-Limonene oxide	-	-	-	0.1	-	-
1137	1137	*trans*-Limonene oxide	-	-	-	0.1	-	-
1139	1141	(*E*)-Myroxide	-	-	-	0.2	-	-
1141	1141	*trans*-Pinocarveol	-	-	-	0.3	0.1	0.1
1142	1142	*trans-p*-Menth-2-en-1-ol	0.2	0.2	0.2	0.4	0.2	0.2
1146	1146	*trans*-Verbenol	-	-	-	0.2	-	-
1155	1156	Camphene hydrate	-	-	-	0.1	-	-
1162	1164	Pinocarvone	-	-	-	0.2	-	-
1168	1167	Benzoic acid	1.9	2.8	4.7	1.7	2.2	2.0
1170	1169	Rosefuran epoxide	-	-	-	0.1	-	-
1172	1171	*p*-Mentha-1,5-dien-8-ol	-	-	-	-	0.1	0.1
1180	1180	Terpinen-4-ol	0.3	0.5	0.3	0.4	0.6	0.3
1187	1185	Cryptone	0.4	0.4	0.9	2.2	0.5	0.2
1188	1188	*p*-Cymen-8-ol	-	-	-	3.7	-	-
1195	1195	α-Terpineol	1.5	1.0	1.3	1.5	1.6	1.3
1197	1196	*cis*-Piperitol	0.1	0.1	0.1	-	0.1	0.1
1197	1195	Myrtenol	-	-	-	0.4	-	-
1203	1202	*cis*-Sabinol	0.3	0.1	0.3	-	0.3	0.5
1207	1205	Verbenone	0.1	0.1	0.1	0.3	0.3	0.1
1209	1208	*trans*-Piperitol	0.1	0.1	0.1	0.1	0.1	tr
1223	1227	4-Isopropylphenol	-	-	-	0.1	-	-
1227	1227	Citronellol	0.1	-	-	-	-	-
1228	1229	Thymyl methyl ether	0.7	1.1	0.7	0.1	0.6	0.1
1244	1242	Cuminal	-	-	-	0.5	-	-
1246	1246	Carvone	-	-	-	0.1	-	-
1256	1254	Piperitone	-	-	-	0.1	-	-
1283	1282	Bornyl acetate	0.1	0.2	tr	0.2	0.1	tr
1288	1287	α-Terpinen-7-al	-	-	-	0.1	-	-
1293	1291	*p*-Cymen-7-ol	-	-	-	0.7	-	-
1321	1320	Methyl geranate	-	-	-	0.3	-	0.1
1324	1318	4-Hydroxycryptone	-	-	-	0.2	-	-
1340	1339	3-Oxo-*p*-Menth-1-en-7-al	-	-	-	0.3	-	-
1347	1348	α-Cubebene	-	0.1	-	-	-	-
1376	1375	α-Copaene	0.1	0.1	tr	0.2	0.1	-
1378	1378	Geranyl acetate	0.1	0.1	0.1	0.1	0.1	0.1
1403	1405	Siberene	tr	0.1	-	-	-	-
1419	1417	(*E*)-β-Caryophyllene	0.1	0.1	0.1	-	0.1	0.1
1430	1430	β-Copaene	tr	tr	-	-	-	-
1452	1451	(*E*)-β-Farnesene	tr	0.1	0.1	0.1	0.1	tr
1455	1454	α-Humulene	0.1	0.1	tr	0.1	0.1	tr
1475	1475	γ-Muurolene	0.1	0.2	0.1	0.3	0.1	tr
1481	1480	Germacrene D	0.2	0.2	0.1	-	0.2	0.2
1489	1489	β-Selinene	0.2	0.2	0.1	0.6	0.2	0.1
1492	1490	γ-Amorphene	tr	0.1	-	-	-	-
1496	1497	α-Selinene	0.2	0.2	0.1	0.4	0.1	0.1
1498	1497	α-Muurolene	0.3	0.3	0.2	0.4	0.3	0.2
1512	1512	γ-Cadinene	0.5	0.7	0.3	1.4	0.3	0.2
1518	1518	δ-Cadinene	1.0	1.3	0.9	0.5	1.0	0.8
1521	1519	*trans*-Calamenene	tr	tr	tr	0.1	tr	tr
1536	1538	α-Cadinene	tr	tr	tr	0.1	tr	tr
1541	1541	α-Calacorene	-	-	-	0.2	-	-
1560	1560	(*E*)-Nerolidol	0.1	0.1	0.1	0.3	0.3	0.3
1576	1574	Germacra-1(10),5-dien-4β-ol	-	-	0.1	-	0.1	0.1
1609	1611	Humulene epoxide II	-	-	-	0.1	-	-
1614	1614	1,10-di-*epi*-Cubenol	tr	0.1	tr	0.1	0.1	tr
1627	1628	1-*epi*-Cubenol	0.2	0.3	0.1	0.3	0.2	0.1
1641	1640	τ-Cadinol	0.5	0.7	0.6	1.1	0.8	0.6
1643	1644	τ-Muurolol	0.5	0.6	0.7	1.1	1.1	0.7
1646	1645	α-Muurolol (=δ-Cadinol)	0.2	0.2	0.2	0.4	0.4	0.3
1655	1655	α-Cadinol	0.7	0.8	1.9	1.8	2.7	2.1
1657	1660	Selin-11-en-4α-ol	0.1	0.1	0.1	0.3	0.2	0.1
1657	1663	*cis*-Calamenen-10-ol	-	-	-	0.5	-	-
1662	1660	*ar*-Turmerone	0.3	0.1	0.2	0.6	0.2	0.1
1664	1671	*trans*-Calamenen-10-ol	-	-	-	0.2	-	-
1667	1668	α-Turmerone	0.2	0.1	0.1	-	0.1	0.1
1678	1676	(9*Z*,12*E*)-Tetradecadien-1-ol	0.1	0.1	0.2	-	0.3	0.6
1700	1699	β-Turmerone (=Curlone B)	0.2	0.1	0.1	-	0.1	tr
1866	1869	Benzyl salicylate	tr	0.1	tr	0.1	-	-
1932	1933	Beyerene	3.2	4.4	0.2	9.1	4.5	4.6
1994	1997	Kaur-15-ene	0.1	0.1	-	0.2	0.1	0.1
2049	2049	Abietatriene	0.1	0.1	0.5	1.5	-	-
2051	2053	Manool	-	-	-	-	0.3	0.2
2082	2086	Abietadiene	0.1	0.1	0.1	-	-	-
		Monoterpene hydrocarbons	82.0	78.9	81.2	54.1	74.3	78.9
		Oxygenated monoterpenoids	4.5	4.2	5.0	14.8	5.1	3.3
		Sesquiterpene hydrocarbons	2.8	3.7	1.9	4.3	2.5	1.6
		Oxygenated sesquiterpenoids	3.0	3.1	4.3	6.9	6.2	4.4
		Diterpenoids	3.5	4.7	0.8	10.8	4.9	5.0
		Benzenoid aromatics	1.9	2.9	4.7	2.0	2.2	2.0
		Others	1.9	2.4	1.6	2.1	4.7	4.4
		Total identified	99.6	99.9	99.5	95.0	99.9	99.6

RI_calc_ = Retention index calculated with respect to a homologous series of *n*-alkanes on a ZB-5ms column [59]. RI_db_ = Reference retention index from the databases [60,61,62,63]. tr = trace (<0.05%).

**Table 4:** The enantiomeric distribution (percent of each enantiomer) of the chiral terpenoids in *Chamaecyparis lawsoniana*.

Compounds	RI_db_	RI_calc_	*C.l.* #1	*C.l.* #2
(+)-α-Thujene	950	n.o.	0.0	0.0
(−)-α-Thujene	951	951	100.0	100.0
(−)-α-Pinene	976	977	19.8	3.1
(+)-α-Pinene	982	981	80.2	96.9
(+)-Sabinene	1021	1018	99.6	99.4
(−)-Sabinene	1030	1031	0.4	0.6
(+)-β-Pinene	1027	1027	100.0	100.0
(−)-β-Pinene	1031	n.o.	0.0	0.0
(−)-Limonene	1073	1077	0.1	0.2
(+)-Limonene	1081	1079	99.9	99.8
(+)-*cis*-Sabinene hydrate	1199	1199	100.0	100.0
(−)-*cis*-Sabinene hydrate	1202	n.o.	0.0	0.0
(+)-*trans*-Sabinene hydrate	1231	1231	96.3	95.0
(−)-*trans*-Sabinene hydrate	1235	1236	3.7	5.0
(+)-Terpinen-4-ol	1297	1293	67.2	72.5
(−)-Terpinen-4-ol	1300	1298	32.8	27.5
(−)-Bornyl acetate	1344	1346	100.0	100.0
(+)-Bornyl acetate	n.a.	n.o.	0.0	0.0
(−)-α-Terpineol	1347	1348	31.8	33.5
(+)-α-Terpineol	1356	1356	68.2	66.5
(−)-δ-Cadinene	1563	n.o.	0.0	0.0
(+)-δ-Cadinene	1576	1575	100.0	100.0

RI_db_ = Retention index from our in-house database based on commercially available compounds available from Sigma-Aldrich and augmented with our own data. RI_calc_ = Calculated retention index based on a series of *n*-alkanes on a Restek B-Dex 325 capillary column. n.o. = not observed. n.a. = no reference compound available.

**Table 5:** The enantiomeric distribution (percent of each enantiomer) of the chiral terpenoids in *Thuja plicata*.

Compounds	RI_db_	RI_calc_	*T.p.* #1	*T.p.* #2	*T.p.* #3
(+)-α-Thujene	950	n.o.	0.0	0.0	0.0
(−)-α-Thujene	951	953	100.0	100.0	100.0
(−)-α-Pinene	976	979	4.5	1.2	1.0
(+)-α-Pinene	982	983	95.5	98.8	99.0
(+)-Sabinene	1021	1020	100.0	100.0	100.0
(−)-Sabinene	1030	n.o.	0.0	0.0	0.0
(+)-β-Pinene	1027	1027	100.0	100.0	100.0
(−)-β-Pinene	1031	n.o.	0.0	0.0	0.0
(−)-Limonene	1073	1075	2.9	2.9	3.3
(+)-Limonene	1081	1081	97.1	97.1	96.7
(+)-*cis*-Sabinene hydrate	1199	1197	97.2	97.6	96.8
(−)-*cis*-Sabinene hydrate	1202	1200	2.8	2.4	3.2
(+)-α-Thujone	1213	n.o.	0.0	0.0	0.0
(−)-α-Thujone	1222	1221	100.0	100.0	100.0
(+)-β-Thujone	1230	1227	100.0	100.0	100.0
(−)-β-Thujone	n.a.	n.o.	0.0	0.0	0.0
(+)-Terpinen-4-ol	1297	1293	75.2	70.3	74.4
(−)-Terpinen-4-ol	1300	1298	24.8	29.7	25.6
(−)-α-Terpineol	1347	1347	35.5	39.4	37.8
(+)-α-Terpineol	1356	1355	64.5	60.6	62.2

RI_db_ = Retention index from our in-house database based on commercially available compounds available from Sigma-Aldrich and augmented with our own data. RI_calc_ = Calculated retention index based on a series of *n*-alkanes on a Restek B-Dex 325 capillary column. n.o. = not observed. n.a. = no reference compound available.

**Table 6:** The enantiomeric distribution (percent of each enantiomer) of the chiral terpenoid components in the foliar essential oil of the *Tsuga heterophylla* from Oregon.

Compounds	RI_db_	RI_calc_	Cascade Range			Coastal Range		
			*T.h.* #1	*T.h.* #2	*T.h.* #3	*T.h.* #4	*T.h.* #5	*T.h.* #6
(−)-α-Pinene	976	976	53.0	73.8	59.3	76.6	44.5	38.3
(+)-α-Pinene	982	981	47.0	26.2	40.7	23.4	55.5	61.7
(−)-Camphene	998	1001	72.1	79.9	75.0	84.9	71.5	62.3
(+)-Camphene	1005	1006	27.9	20.1	25.0	15.1	28.5	37.7
(+)-Sabinene	1021	1021	100.0	100.0	100.0	59.6	100.0	83.2
(−)-Sabinene	1030	1028	0.0	0.0	0.0	40.4	0.0	16.8
(+)-β-Pinene	1027	1027	2.2	1.9	2.1	3.6	3.5	8.3
(−)-β-Pinene	1031	1030	97.8	98.1	97.9	96.4	96.5	91.7
(−)-α-Phellandrene	1050	1051	3.5	11.4	8.2	n.o.	7.1	1.7
(+)-α-Phellandrene	1053	1052	96.5	88.6	91.8	n.o.	92.9	98.3
(−)-Limonene	1073	1074	67.6	81.3	77.8	92.6	76.5	85.8
(+)-Limonene	1081	1081	32.4	18.7	22.2	7.4	23.5	14.2
(−)-β-Phellandrene	1083	1083	76.7	93.5	91.9	95.2	90.0	36.8
(+)-β-Phellandrene	1089	1088	23.3	6.6	8.1	4.8	10.0	63.2
(−)-Linalool	1228	1228	68.2	16.7	60.8	48.1	30.2	54.7
(+)-Linalool	1231	1231	31.8	83.3	39.2	51.9	69.8	45.3
(+)-Terpinen-4-ol	1297	1297	42.2	31.8	38.0	30.9	34.1	48.2
(−)-Terpinen-4-ol	1300	1300	57.8	68.2	62.0	69.1	65.9	51.8
(−)-Bornyl acetate	1344	1345	100.0	100.0	100.0	100.0	100.0	100.0
(+)-Bornyl acetate	n.a.	n.o.	0.0	0.0	0.0	0.0	0.0	0.0
(−)-α-Terpineol	1347	1347	76.5	82.7	85.7	89.6	77.6	62.9
(+)-α-Terpineol	1356	1353	23.5	17.3	14.3	10.4	22.4	37.1
(+)-Germacrene D	1519	n.o.	0.0	0.0	0.0	n.o.	0.0	0.0
(−)-Germacrene D	1522	1524	100.0	100.0	100.0	n.o.	100.0	100.0
(−)-δ-Cadinene	1563	n.o.	0.0	0.0	0.0	0.0	0.0	0.0
(+)-δ-Cadinene	1576	1577	100.0	100.0	100.0	100.0	100.0	100.0
(−)-(*E*)-Nerolidol	1677	1677	32.1	17.3	20.2	15.6	37.0	47.4
(+)-(*E*)-Nerolidol	1680	1679	67.9	82.7	79.8	84.4	63.0	52.6

RI_db_ = Retention index from our in-house database based on commercially available compounds available from Sigma-Aldrich and augmented with our own data. RI_calc_ = Calculated retention index based on a series of *n*-alkanes on a Restek B-Dex 325 capillary column. n.o. = not observed. n.a. = no reference compound available.

**Table 7:** The enantiomeric distribution (percent of each enantiomer) of the chiral terpenoids in members of the Pinaceae and Cupressaceae.

	α-Pinene		Camphene		Sabinene		β-Pinene		Limonene		β-Phellandrene		Linalool		Terpinen-4-ol		α-Terpineol		
Pinaceae	(+)	(−)	(+)	(−)	(+)	(−)	(+)	(−)	(+)	(−)	(+)	(−)	(+)	(−)	(+)	(−)	(+)	(−)	Ref.
*Abies concolor*	72	28	4	96	n.o.	n.o.	1	99	11	89	1	99	71	29	39	61	14	86	[68]
*Abies lasiocarpa* var. *lasiocarpa*	24	76	3	97	n.o.	n.o.	1	99	6	94	0	100	30	70	31	69	n.o	n.o	[49]
*Abies procera*	43	57	0	100 ^a^	n.o.	n.o.	2	98	0	100	2	98	n.o.	n.o.	48	52	8	92	[58]
*Picea engelmannii* subsp. *engelmannii*	37	63	7	93	n.o.	n.o.	4	96	5	95	11	89	32	68	44	56	47	53	[49]
*Picea pungens*	36	64	7	93	100	0	3	97	4	96	7	93	26	74	42	58	48	52	[69]
*Pinus contorta* subsp. *contorta*	27	73	n.o.	n.o.	n.o.	n.o.	0	100	13	87	1	99	n.o.	n.o.	53	47	35	65	[70]
*Pinus contorta* subsp. *latifolia*	13	87	21	79	n.o.	n.o.	2	98	12	88	1	99	20	80	44	56	6	94	[49]
*Pinus contorta* subsp. *murrayana*	20	80	n.o.	n.o.	n.o.	n.o.	2	98	0	100	1	99	0	100	40	60	3	97	[58]
*Pinus edulis*	64	36	29	71	18	82	3	97	30	70	1	99	34	66	37	63	24	76	[71]
*Pinus flexilis*	5	95	2	98	100	0	3	97	33	67	3	97	n.o.	n.o.	43	57	9	91	[70]
*Pinus monophylla*	70	30	43	57	n.o.	n.o.	3	97	31	69	1	99	n.o.	n.o.	34	66	26	74	[71]
*Pinus ponderosa* var. *ponderosa*	27	73	22	78	n.o.	n.o.	2	98	40	60	1	99	9	91	36	64	3	97	[70]
*Pseudotsuga menziesii* var. *glauca*	18	82	2	98	2	98	2	98	18	82	3	97	7	93	34	66	17	83	[49]
*Tsuga heterophylla*	n.o.	n.o.	n.o.	n.o.	n.o.	n.o.	n.o.	n.o.	0	100	0	100	n.o.	n.o.	30	70	13	87	[58]
*Tsuga heterophylla*	42	58	26	74	92	8	4	96	20	80	19	81	54	46	37	63	21	79	t.w.
**Cupressaceae**																			
*Chamaecyparis lawsoniana*	89	11	n.o	n.o.	99	1	100	0	99	1	n.o.	n.o.	n.o.	n.o.	70	30	67	33	t.w.
*Juniperus horizontalis*	81	19	63	37	100	0	100	0	83	17	31	69	61	39	67	33	51	49	[72]
*Juniperus osteosperma*	99	1	93	7	100	0	100	0	98	2	n.o	n.o	n.o.	n.o.	67	33	100	0	[73]
*Juniperus scopulorum*	92	8	52	48	100	0	100	0	90	10	47	53	88	12	53	47	54	46	[72]
*Thuja plicata*	83	17	n.o.	n.o.	100	0	85	15	96	4	n.o	n.o	n.o.	n.o.	74	26	68	32	[49]
*Thuja plicata*	98	2	n.o.	n.o.	100	0	100	0	97	3	n.o	n.o	n.o.	n.o.	73	27	62	38	t.w.

^a^ Enantiomer misassigned in the original report. n.o. = not observed. t.w. = this work (mean values).

**Table 8:** The collection and hydrodistillation details for the *Chamaecyparis lawsoniana*, *Thuja plicata*, and *Tsuga heterophylla* foliar essential oils.

Sample	Date	Collection Site	Mass Foliage	Mass Essential Oil	Yield, Color
*C. lawsoniana* #1	15-Apr-23	45°2′16″ N, 123°48′29″ W, 116 m asl	82.74 g	1.5694 g	1.897%, pale yellow
*C. lawsoniana* #2	15-Apr-23	45°2′19″ N, 123°48′30″ W, 117 m asl	158.78 g	3.6999 g	2.330%, pale yellow
*T. plicata* #1	14-Apr-23	45°20′58″ N, 121°59′39″ W, 362 m asl	81.66 g	0.6684 g	0.814%, pale yellow
*T. plicata* #2	14-Apr-23	45°20′58″ N, 121°59′46″ W, 359 m asl	91.83 g	0.6932 g	0.755%, colorless
*T. plicata* #3	14-Apr-23	45°20′58″ N, 121°59′51″ W, 359 m asl	54.91 g	0.5653 g	1.030%, colorless
*T. heterophylla* #1	14-Apr-23	45°20′58″ N, 121°59′40″ W, 362 m asl	70.39 g	3.7160 g	5.279%, colorless
*T. heterophylla* #2	14-Apr-23	45°20′58″ N, 121°59′44″ W, 360 m asl	62.83 g	3.9040 g	6.214%, colorless
*T. heterophylla* #3	14-Apr-23	45°20′59″ N, 121°59′51″ W, 359 m asl	50.53 g	3.1256 g	6.186%, colorless
*T. heterophylla* #4	16-Apr-23	45°2′15″ N, 123°48′29″ W, 114 m asl	78.89 g	4.3224 g	5.479%, colorless
*T. heterophylla* #5	16-Apr-23	45°2′17″ N, 123°48′30″ W, 117 m asl	95.21 g	5.9732 g	6.274%, colorless
*T. heterophylla* #6	16-Apr-23	45°2′18″ N, 123°48′30″ W, 117 m asl	61.30 g	4.7489 g	7.747%, colorless

## Data Availability

All data are available in the article.
